# Changes in Dopamine Transmission in the Nucleus Accumbens Shell and Core during Ethanol and Sucrose Self-Administration

**DOI:** 10.3389/fnbeh.2017.00071

**Published:** 2017-05-01

**Authors:** Valentina Bassareo, Flavia Cucca, Roberto Frau, Gaetano Di Chiara

**Affiliations:** ^1^Department of Biomedical Sciences, University of CagliariCagliari, Italy; ^2^Institute of Neuroscience, Cagliari Section, University of CagliariCagliari, Italy; ^3^National Institute of Neuroscience CNR, University of CagliariCagliari, Italy

**Keywords:** mesolimbic system, self-administration, sucrose, ethanol, dopamine, shell, core

## Abstract

Ethanol, like other substances of abuse, preferentially increases dopamine (DA) transmission in the rat nucleus accumbens (NAc) following passive administration. It remains unclear, however, whether ethanol also increases NAc DA transmission following operant oral self-administration (SA). The NAc is made-up of a ventro-medial compartment, the shell and a dorso-lateral one, the core, where DA transmission responds differentially following exposure to drugs of abuse. Previous studies from our laboratory investigated changes in dialysate DA in the NAc shell and core of rats responding for sucrose pellets and for drugs of abuse. As a follow up to these studies, we recently investigated the changes in NAc shell and core DA transmission associated to oral SA of a 10% ethanol solution. For the purpose of comparison with literature studies utilizing sucrose + ethanol solutions, we also investigated the changes in dialysate DA associated to SA of 20% sucrose and 10% ethanol + 20% sucrose solutions. Rats were trained to acquire oral SA of the solutions under a Fixed Ratio 1 (FR1) schedule of nose-poking. After training, rats were monitored by microdialysis on three consecutive days under response contingent (active), reward omission (extinction trial) and response non-contingent (passive) presentation of ethanol, sucrose or ethanol + sucrose solutions. Active and passive ethanol administration produced a similar increase in dialysate DA in the two NAc subdivisions, while under extinction trial DA increased preferentially in the shell compared to the core. Conversely, under sucrose SA and extinction DA increased exclusively in the shell. These observations provide unequivocal evidence that oral SA of 10% ethanol increases dialysate DA in the NAc, and also suggest that stimuli conditioned to ethanol exposure contribute to the increase of dialysate DA observed in the NAc following ethanol SA. Comparison between the pattern of DA changes detected in the NAc subdivisions under sucrose and ethanol SA likewise suggests that the NAc shell and core DA play different roles in sucrose as compared to ethanol reinforcement.

## Introduction

Drinking of alcohol plays an important social role in numerous cultures, with alcoholic beverages being legally available throughout the majority of countries worldwide. However, alcohol consumption may lead to dependance; indeed, the high prevalence of alcoholism in the general population represents a major social and health issue, and considerable effort has been devoted on clarifying the neurobiological bases of this condition.

A large number of studies have focused on the ability of ethanol to stimulate *in vivo* dopamine (DA) transmission in the nucleus accumbens (NAc) of rats and mice (Imperato and Di Chiara, 1986; Weiss et al., 1993; Gonzales and Weiss, 1998; Melendez et al., 2002; Doyon et al., 2003, 2005; Tang et al., 2003; for review see Gonzales et al., 2004). The NAc, however, is not a homogeneous structure, being made up of a ventro-medial subdivision, the shell and a dorso-lateral one, the core, possessing different input-out connections and functions. Drugs of abuse such as cocaine, amphetamine, morphine, heroin, THC, MDMA and nicotine preferentially activate shell DA transmission following response non-contingent (passive) as well as response-contingent exposure (Pontieri et al., 1995, 1996; Tanda et al., 1997; Lecca et al., 2006a,b, 2007a,b; Aragona et al., 2008).

While the ability of response non-contingent ethanol to increase dialysate DA in the NAc, and in particular the shell, is well established (Di Chiara and Imperato, 1985; Imperato and Di Chiara, 1986; Di Chiara et al., 1996; Bassareo et al., 2003; Howard et al., 2008), it remains unclear whether the same also applies to response-contingent oral administration (self-administration, SA). Previous microdialysis studies in rats self-administering oral ethanol solutions have estimated the changes in DA transmission in the NAc by taking as basal (100%) values the levels of dialysate DA in samples collected in the rat’s home cage (Weiss et al., 1993; Gonzales and Weiss, 1998; Doyon et al., 2003, 2005; Howard et al., 2009). However, transfer to the Skinner box equipped for microdialysis monitoring was found to increase *per se* dialysate DA in the NAc (Weiss et al., 1993; Gonzales and Weiss, 1998; Doyon et al., 2003, 2005; Howard et al., 2009). Accordingly, as highlighted by Gonzales and Weiss (1998), it is unclear to what extent the increase in dialysate DA observed in the NAc under ethanol SA is affected by the DA-activating influence of transfer from the home cage to the Skinner box (Weiss et al., 1993; Gonzales and Weiss, 1998; Doyon et al., 2003, 2005; Howard et al., 2009). On the other hand, in studies from the same group that distinguished the NAc shell from core, no increase of DA was observed, except for microdialysis probe placements at the border between the shell and core, an area that the same authors indicate as “shore” but devoid of anatomical and physiological identity (Howard et al., 2008). A further point of uncertainty in the paradigm adopted in the above studies arises from the failure of sucrose SA to increase dialysate DA in any subdivision of the NAc (Howard et al., 2009). This observation is in contrast with a large number of studies showing that sucrose SA increases dialysate DA, depending on the experimental conditions, specifically in the NAc shell, or in both the shell and core (Bassareo and Di Chiara, 1997, 1999; Brown et al., 2011; Cacciapaglia et al., 2012; Bassareo et al., 2015a,b,c).

This premise shows that it is still unclear if indeed oral ethanol SA increases DA transmission in the NAc, as estimated by microdialysis. In an attempt to shed light on this issue we compared changes in dialysate DA in the shell and core of rats trained to respond for oral ethanol (10% solution), sucrose (20% solution) and 10% ethanol in 20% sucrose solution. Rats were tested on three consecutive days under operant (active), extinction trial and passive ethanol presentation. This procedure has previously been used by our group in studies of sucrose pellet reinforcement (Bassareo et al., 2015a,b,c).

## Materials and Methods

### Animals

Male Sprague-Dawley rats (Harlan Italy, Udine, Italy) weighing 250–275 g were housed in group of six per cage (h:20 cm × w:38 cm × l:59 cm) with standard chow (Stefano Morini, S. Polo D’Enza, RE, Italy) and water available *ad libitum*, for at least 1 week in the central animal room under constant temperature (23°C), humidity (60%) and a 12 h light/dark cycle (light on from 8 a.m. to 8 p.m.).

This study was carried out in accordance with the guidelines for care and use of experimental animals of the European Communities Council (2010/63/UE L 276 20/10/2010) and with Italian law (DL: 04.03.2014, N°26), and approved by the Organism for animal care of University of Cagliari (OPBA). Every effort was made to minimize suffering and reduce the numbers of animals used.

### Surgery

Rats were anesthetized as previously reported by our group (Bassareo et al., 2015a). A guide cannula (Plasticone, Roanoke, VA, USA; Ø: 0,022 mm) was stereotaxically and unilaterally implanted, randomly in the left or in the right hemisphere according to the following coordinates: NAc shell (A: 2.0 mm; L: 1 mm from bregma, V: −3.6 mm from dura), NAc core (A: 1.6 mm; L: 1.9 mm from bregma, V: −3.4 mm from dura) according to the atlas of Paxinos and Watson (1998). Guide cannulae were plugged with a dummy cannula.

After surgery, rats were housed in individual cages (45 × 21 × 24 cm) under the same conditions mentioned above. Rats were left to recover for 10 days, and Gentamicin sulfate (40 mg/Kg s.c.) was administered over the first 5 days. Rats were handled once daily for 5 min throughout the training period to habituate them to contact with the operator and all procedures.

After recovery rats were fed daily with 20 g standard chow (Stefano Morini, S. Polo D’ Enza, RE, Italy) and their weight maintained at approx. 95% their *ad libitum* weight. Water was available *ad libitum* throughout the duration of experiments.

### Microdialysis

#### Probe Preparation

Microdialysis probes were prepared according to the method of Lecca et al. (2006a,b) and reported by us (Bassareo et al., 2015c), using AN69 membrane (Hospal Dasco, Italy). The length of the dialyzing portion of the probe was 1.5 mm. A new probe was used for each experimental session.

#### Microdialysis Experiments

At the beginning of each microdialysis session, microdialysis probes were connected to an infusion pump and perfused with Ringer’s solution (147 mM NaCl, 4 mM KCl, 2.2 mM CaCl_2_; see Lecca et al., 2006a on the use of 2.2 mM Ca^2+^ in the Ringer) at a constant rate of 1 μl/min. The dummy cannula was removed and the microdialysis probe inserted through the guide cannula. Dialysate samples (10 μl) were taken every 10 min and injected without purification into either a high-performance liquid chromatograph (HPLC) or an ultra HPLC (UHPLC; ALEXYS Neurotransmitter analyzer, Antec).

The HPLC was equipped with a reverse phase column (LC-18 DB, 15 cm, 5 μm particle size, Supelco) and a coulometric detector (ESA, Coulochem II, Bedford, MA, USA) to quantify DA. The first electrode of the detector was set at +125 mV (oxidation) and the second at −175 mV (reduction). The composition of the mobile phase was: 50 mM NaH_2_PO_4_, 0.1 mM Na_2_-EDTA, 0.5 mM n-octyl sodium sulfate, 15% (v/v) methanol, pH 5.5 (obtained adding Na_2_HPO_4_). Under these conditions, sensitivity of the assay for DA was 5 fmol/sample. The UHPLC was equipped with a NeuroSep (C18 110, 1.0 × 100 mm, 1.7 μm) column and an electrochemical amperometric detector (DECADE II SCC). Composition of the mobile phase was: 100 mM phosphoric acid, 100 mM citric acid, 0.1 mM EDTA, acetonitrile 8% v/v, 3 mM. Using these conditions sensitivity of the assay for DA was 5 fmol/sample. At the end of each microdialysis session the probe was removed and the guide cannula was once again plugged with a sterilized dummy cannula.

#### Sucrose

The sucrose solution (20%, w/v) was obtained using granulated sugar (SADAM S.p.A., Villasor, Cagliari, Italy) and tap water.

#### Ethanol

The 10% ethanol solution (w/v) was obtained using 95° ethanol (Farmitalia Carlo Erba, Milan, Italy) and tap water or 20% sucrose solution.

#### Training

Ten days after guide cannula implant, rats were trained every day for 3 weeks, with the exception of weekends. Sessions lasted 1 h and took place between 9.00 am and 2.00 pm in acoustically isolated and ventilated operant cages (Coulbourn Instruments, Allentown, NJ, USA). Two nose-poke holes were placed on one wall, 2 cm from the cage floor. The active nose-poke was illuminated by a green-yellow light, and the inactive one by a red light. The liquid solenoid valve for solution delivery was inserted between the nose-pokes holes and a light placed above it. A loudspeaker emitting a tone of 4500 Hz was located on the same wall.

Rats were trained to respond for 20% sucrose solution, for 10% ethanol in 20% sucrose solution, or for 10% ethanol solution under a Fixed Ratio 1 (FR1) schedule. Each active nose poke = 0.28 ml of each solution. The number of nose-pokes performed and rewards earned were recorded by Graphic State 2 software, Coulbourn Instruments, Whitehall, PA, USA.

Each 1-h session was made up of a cyclic alternation of three phases:

- Phase 1, lasting 15 s, during which the house light and nose poke lights were turned on and a tone was activated to signal reward availability. Failure to respond correctly for more than 15 s resulted in switch off of visual and auditory cues and start of phase 3, bypassing phase 2.
- Phase 2: 0.28 ml of 20% sucrose solution, or 10% ethanol in 20% sucrose solution, or 10% ethanol solution were delivered into the valve and the light above the valve was switched on. After 5 s phase 3 was initiated.
- Phase 3 (time out): all cues were turned off and reward was not available for 7 s.

A significant difference (*p* < 0.05) between active and inactive nose pokes for at least five consecutive sessions (1 session × day) was taken as criterion for full training.

#### Microdialysis after Training

Following completion of training rats were tested in three daily microdialysis sessions performed on three consecutive days. Sessions were started as soon as DA basal dialysate levels had stabilized (i.e., after approximately 1 h).

First day: FR1 responding for 20% sucrose solution, or for 10% ethanol in 20% sucrose solution, or for 10% ethanol solution.

Second day: responding under extinction i.e., by substituting tap water for ethanol or sucrose solutions, but in the presence of all the stimuli preceding and following each response.

Third day: non-contingent presentation of 20% sucrose, 10% ethanol in 20% sucrose or 10% ethanol solutions at the same mean rate of operant responding in the absence of discriminative cues signaling reward availability.

#### Histology

**Figure 1:**
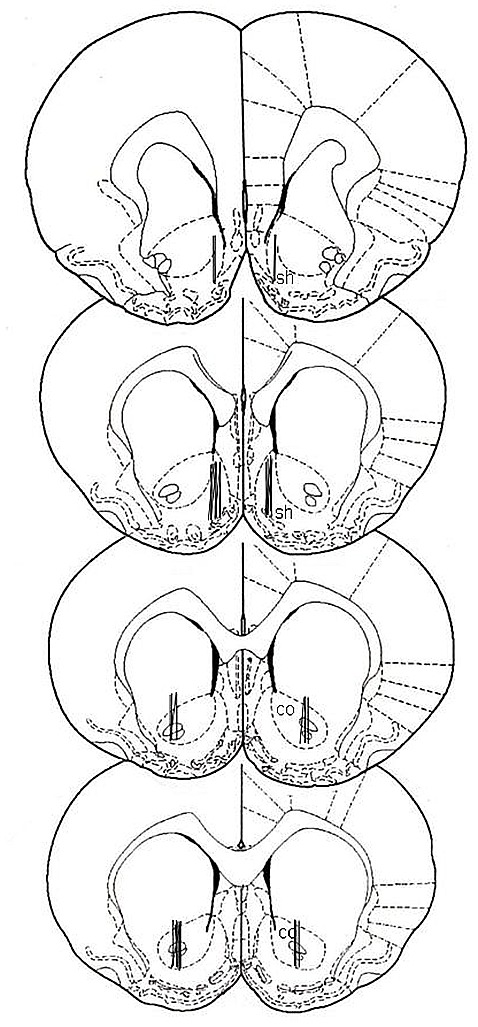
**Localization of dialysis probes (dialysis portion) within the nucleus accumbens (NAc) shell and core according to Paxinos and Watson (1998).** sh, shell; co, core.

At the end of all experimental procedures, rats were anesthetized as previously reported (Bassareo et al., 2015b), and the brains removed and postfixed for 5 days. Brains were cut in 100-μm-thick serial coronal slices on a Vibratome (Technical Products International, Saint Louis, MO, USA) to establish the location of dialysis probes. Probe location was reconstructed and referred to the atlas of Paxinos and Watson (1998; Figure 1).

#### Statistics

Statistical analysis was carried out using Statistica for Windows. Basal dialysate DA was calculated as the mean of the last three consecutive samples differing by no more than 10%, collected during the 60-min period preceding each experimental session. Inter-group comparison of basal dialysate DA values, expressed as femtomoles per 10-μl dialysate, was performed using one or two-way ANOVA. Changes in dialysate DA were expressed as percentage of respective baseline values and were analyzed using two-way ANOVA for repeated measures. Cumulative nose-pokes registered during training and during experiments were analyzed by three-way ANOVA. The number of nose-pokes registered during each 10 min sampling were compared by two-way ANOVA between rats whose probes were implanted either in the shell or the core and combined in the absence of statistical differences. The amount of solutions (in ml) and ethanol intake (in g/Kg) were analyzed using two-way ANOVA for repeated measures.

The results of treatments showing significant overall changes were subjected to *post hoc* Tukey’s test, with statistical significance set at *p* < 0.05.

## Results

Basal values of dialysate DA (fmoles/sample, means ± SEM) corresponded to 46 ± 3 (*N* = 43) in the shell and 48 ± 3 (*N* = 48) in the core, with no differences between the two areas (*F*_(1,89)_ = 3.17; *p* = 0.08). Values obtained were consistent across all groups. Accordingly, two-way ANOVA of basal dialysate DA obtained from the different groups revealed no significant differences between reward utilized or microdialysis sessions either in the shell (*F*_reward 2,34_ = 0.02; *p* = 0.98; *F*_sessions 2,34_ = 0.60; *p* = 0.55; *F*_reward × sessions 4,34_ = 0.34; *p* = 0.85) or the core (*F*_reward 2,39_ = 0.001; *p* = 1; *F*_sessions 2,39_ = 0.29; *p* = 0.75; *F*_reward × sessions 4,39_ = 0.12; *p* = 0.97). The lack of statistically significant differences in absolute basal levels between groups justifies the subsequent analysis of changes in dialysate DA as % of baseline.

### Responding for 20% Sucrose

#### Training

**Figure 2:**
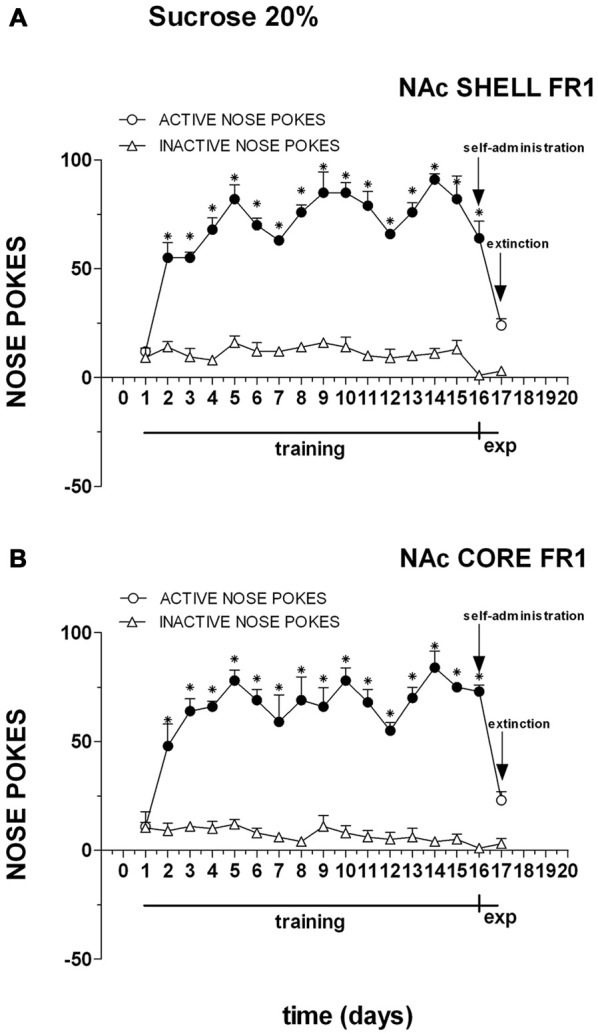
**Cumulative active (circles) and inactive (triangles) nose-pokes during training to respond for 20% sucrose under an fixed ratio 1 (FR1) schedule and during dialysis experiments.** Data are means ± SEM of the results obtained in rats implanted with guide cannulas in the NAc shell **(A)** (*N* = 4) and rats in the NAc core **(B)** (*N* = 4). Filled symbols, *p* < 0.05 vs. 1st day; *p < 0.05 vs. inactive nose pokes.

Figure 2 shows the number of cumulative active and inactive nose pokes performed during training for FR1 responding for sucrose, and on the first and second experiment days. Nose-poking increased selectively on the active hole.

Three-way ANOVA showed a main effect of nose poke (*F*_(1,12)_ = 529.3; *p* < 0.01), days (*F*_(16,192)_ = 53.7; *p* < 0.01) and a nose poke × days interaction (*F*_(16,192)_ = 47.3; *p* < 0.01). Tukey’s test revealed no differences in the temporal profile of acquisition between the shell- and core-implanted groups.

**Figure 3:**
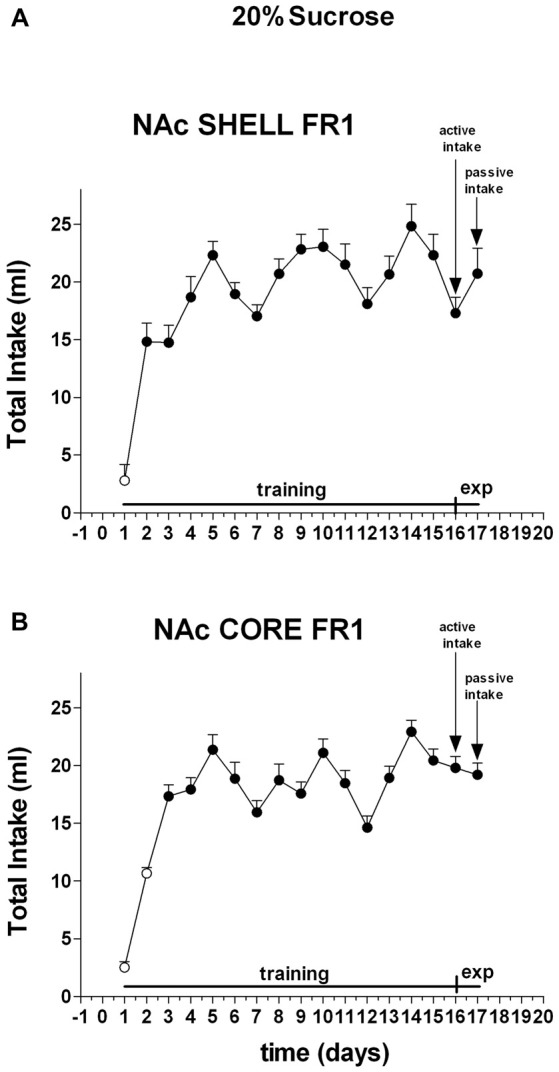
**Total intake expressed in ml of 20% sucrose solution.** Data are means ± SEM of the results obtained in rats implanted with guide cannulas in the NAc shell **(A)** (*N* = 4) and rats in the NAc core **(B)** (*N* = 4). Filled symbols, *p* < 0.05 vs. 1st day.

Two-way ANOVA of the amount of solution (ml) consumed by rats showed a main effect of days (*F*_(16,80)_ = 28.4; *p* < 0.01). *Post hoc* test revealed that the amount of 20% sucrose solution (ml) consumed by rats increased selectively during the training period, but did not reveal any difference between shell- and core-implanted rats (Figure 3).

Monitoring dialysate DA in FR1 trained rats.

#### Operant Session

**Figure 4:**
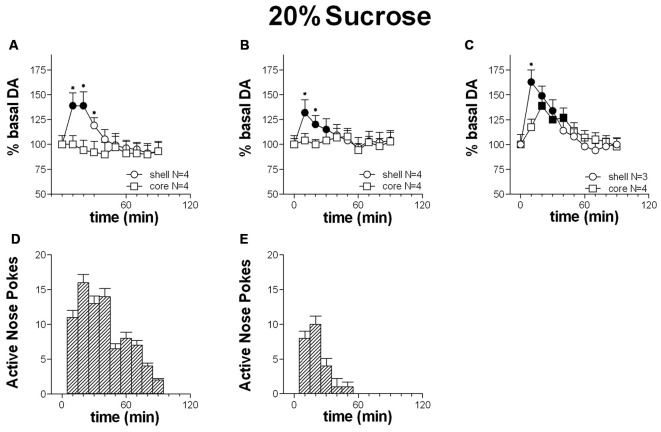
**Time-course of dialysate dopamine (DA) in the NAc shell (circles) and core (squares) and active nose pokes (Bars) every 10 min under FR1 responding for 20% sucrose solution (A,D)**, extinction **(B,E)** and non-contingent sucrose presentation **(C)**. Data are means ± SEM of the results, expressed as % of basal values, obtained in the number (*N*) of rats indicated in the figure. Filled symbols: *p* < 0.05 vs. basal values; *p < 0.05 vs. values obtained in the core.

Figures 4A,D shows the time-course of dialysate DA in the NAc shell and core and the number of active nose-pokes performed during FR1 responding for 20% sucrose solution.

Responding for 20% sucrose solution increased dialysate DA in the shell alone. Core DA remained unchanged throughout the session. A peak in shell DA was displayed at the first sample, and returned to basal levels at the 3rd sample. Responding remained high at four additional samples taken subsequent to the increase of DA in the shell.

Two-way ANOVA showed an effect of area (*F*_(1,6)_ = 32.1; *p* < 0.01), time (*F*_(9,54)_ = 44.5; *p* < 0.01) and an area × time interaction (*F*_(9,54)_ = 32.4; *p* <= 0.01). *Post hoc* test revealed an increase of dialysate DA in the NAc shell but not in the core.

#### Extinction Session

Figures 4B,E shows the time-course of dialysate DA in the NAc shell and core and of active nose-pokes under extinction in the presence of cues signaling sucrose availability and associated to sucrose delivery.

Responding under extinction was associated to a selective increase of DA in the shell. NAc core DA remained unchanged throughout the entire session. A peak in shell DA was displayed at the first sample, and had returned to baseline levels by the 4th sample.

Responding was high for the three samples in the presence of increased DA in the shell.

Two-way ANOVA showed an effect of area (*F*_(1,6)_ = 11.5; *p* = 0.014), time (*F*_(9,54)_ = 30.7; *p* < 0.01) and an interaction area × time (*F*_(9,54)_ = 15.4; *p* < 0.01). *Post hoc* test showed an increase of DA in the NAc shell but not in the core.

#### Non-Contingent 20% Sucrose Solution

Figure 4C shows the time-course of DA in the NAc shell and core following non-contingent 20% sucrose solution presentation.

In contrast to responding for sucrose and to extinction, non-contingent sucrose presentation was associated with an increase of dialysate DA in both the NAc shell and core.

Shell DA peaked at the first sample and had returned to basal values by the 5th sample; Core DA peaked at the 2nd sample and had returned to baseline by the 4th sample.

Two-way ANOVA showed an effect of time (*F*_(9,45)_ = 48.9; *p* < 0.01) and an interaction area × time (*F*_(9,45)_ = 11.8; *p* < 0.01). *Post hoc* test showed an earlier increase in DA in the NAc shell than in the core.

### 10% Ethanol in 20% Sucrose

#### Training

**Figure 5:**
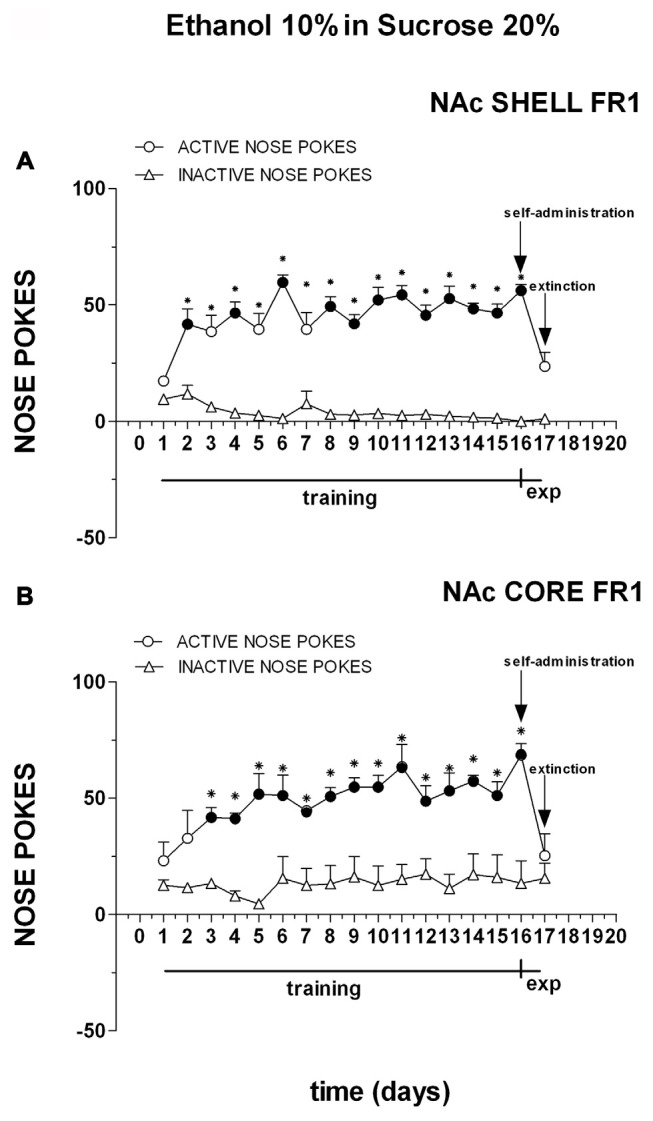
**Cumulative active (circles) and inactive (triangles) nose-pokes during training for FR1 responding for 10% ethanol in 20% sucrose solution and during dialysis experiments.** Data are means ± SEM of the results obtained in rats implanted with guide cannulas in the NAc shell **(A)** (*N* = 6) and rats in the NAc core **(B)** (*N* = 6). Filled symbols, *p* < 0.05 vs. 1st day; *p < 0.05 vs. inactive nose pokes.

Figure 5 shows the number of cumulative active and inactive nose pokes performed during training for FR1 responding for ethanol and sucrose solution, and on the first and the second experiment days. Nose-poking increased selectively on the active hole.

Three-way ANOVA showed a main effect of nose poke (*F*_(1,18)_ = 152.9; *p* < 0.01), days (*F*_(16,288)_ = 7.6; *p* < 0.01) and a nose poke × days interaction (*F*_(16,288)_ = 10.0; *p* < 0.01). Tukey’s test revealed no differences in the temporal profile of acquisition between the shell- and core-implanted groups.

**Figure 6:**
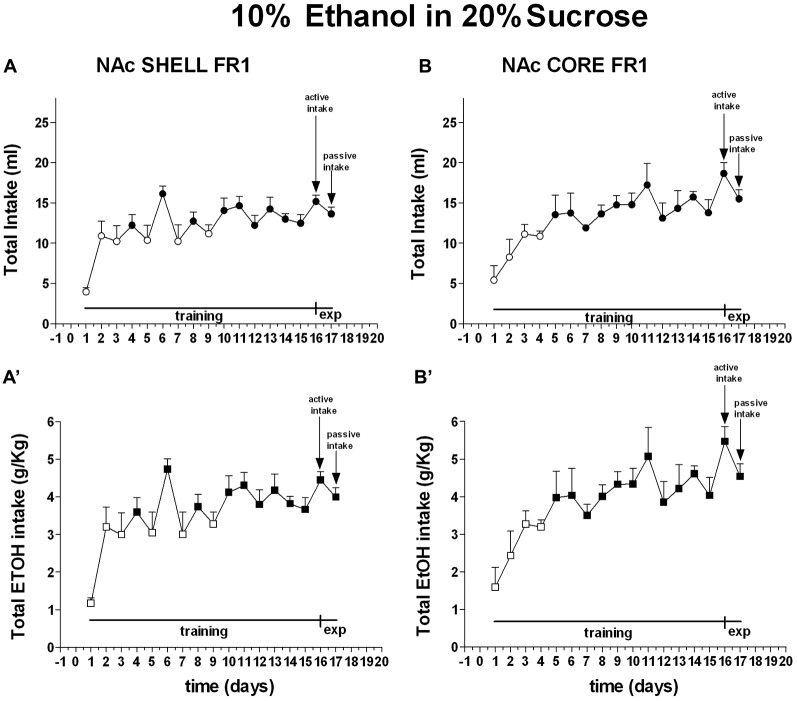
**Total intake expressed as ml of 10% ethanol in 20% sucrose solution and g/Kg of ethanol.** Data are means ± SEM of the results obtained in rats implanted with guide cannulas in the NAc shell (**A**:ml; **A′**:g/Kg; *N* = 6) and rats in the NAc core (**B**:ml; **B′**:g/Kg; *N* = 6). Filled symbols, *p* < 0.05 vs. 1st day.

Two-way ANOVA of the amount of solution (ml) consumed by rats showed a main effect of days (*F*_(16,128)_ = 9.76; *p* < 0.01). *Post hoc* test revealed that the amount of 10% ethanol in 20% sucrose solution (ml) consumed by rats increased selectively during the training period but did not reveal any difference between shell- and core-implanted rats (Figure 6).

Two-way ANOVA of the amount of ethanol (g/Kg) consumed by rats showed a main effect of days (*F*_(16,128)_ = 9.72; *p* < 0.01). *Post hoc* test revealed that the amount of ethanol (g/Kg) consumed by rats increased selectively during the training period but did not reveal any difference between shell- and core-implanted rats (Figure 6).

#### Operant Session

**Figure 7:**
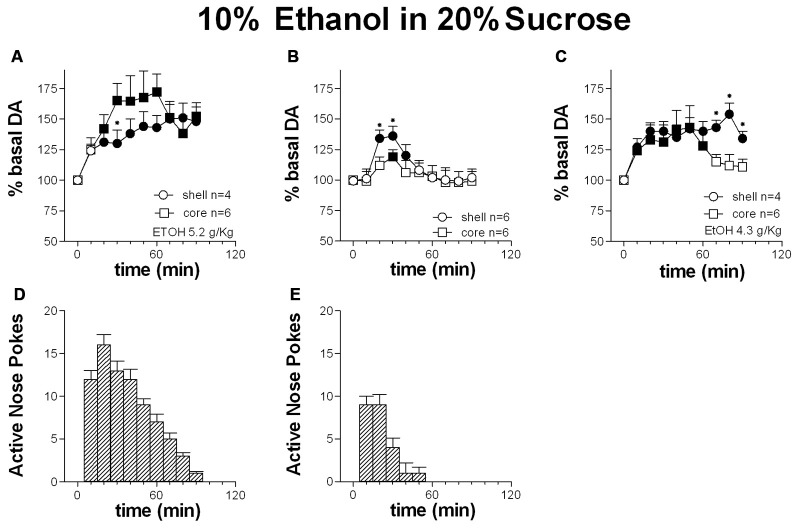
**Time-course of dialysate DA in the NAc shell (circles) and core (squares) and active nose pokes (Bars) every 10 min under FR1 responding for 10% ethanol in 20% sucrose solution (A,D)**, extinction **(B,E)** and non-contingent 10% ethanol in 20% sucrose solution presentation **(C)**. Data are means ± SEM of the results, expressed as % of basal values, obtained in the number (*N*) of rats indicated in the figure. Filled symbols: *p* < 0.05 vs. basal values; *p < 0.05 vs. values obtained in the core.

Figures 7A,D shows the time-course of dialysate DA in the NAc shell and core and the number of active nose-pokes performed during FR1 responding for 10% ethanol in 20% sucrose solution.

Responding for 10% ethanol in 20% sucrose solution increased dialysate DA in both the shell and the core. Responding remained high throughout the session and in the presence of increased DA in the two areas.

Two-way ANOVA showed an effect of area (*F*_(1,8)_ = 5.7; *p* = 0.04), time (*F*_(9,72)_ = 26.8; *p* < 0.01) and an area × time interaction (*F*_(9,72)_ = 5.3; *p* < 0.01). *Post hoc* test revealed a higher increase of DA in the NAc core than in the shell.

#### Extinction Session

Figures 7B,E shows the time-course of dialysate DA in the NAc shell and core and of active nose-pokes under extinction in the presence of cues signaling reward availability and associated to reward delivery. Responding under extinction was associated to an increase of DA in both the shell and the core. Responding was high over the first 30 min.

Two-way ANOVA showed an effect of area (*F*_(1,10)_ = 12.6; *p* < 0.01), time (*F*_(9,90)_ = 30.3; *p* < 0.01) and an interaction area × time (*F*_(9,90)_ = 4.48; *p* < 0.01). *Post hoc* test revealed a higher increase in DA in the NAc shell than in the core.

#### Non-Contingent 10% Ethanol in 20% Sucrose

Figure 7C shows the time-course of DA in the NAc shell and core following the non-contingent presentation of 10% ethanol in 20% sucrose solution.

Non-contingent ethanol-sucrose presentation was associated with an increase in dialysate DA both in the NAc shell and core.

Two-way ANOVA showed an effect of area (*F*_(1,8)_ = 10.9; *p* = 0.01), time (*F*_(9,72)_ = 32.6; *p* < 0.01) and an interaction area × time (*F*_(9,72)_ = 12.5; *p* < 0.01). *Post hoc* test revealed a prolonged increase of DA in the NAc shell compared with the core.

### 10% Ethanol

#### Training

**Figure 8:**
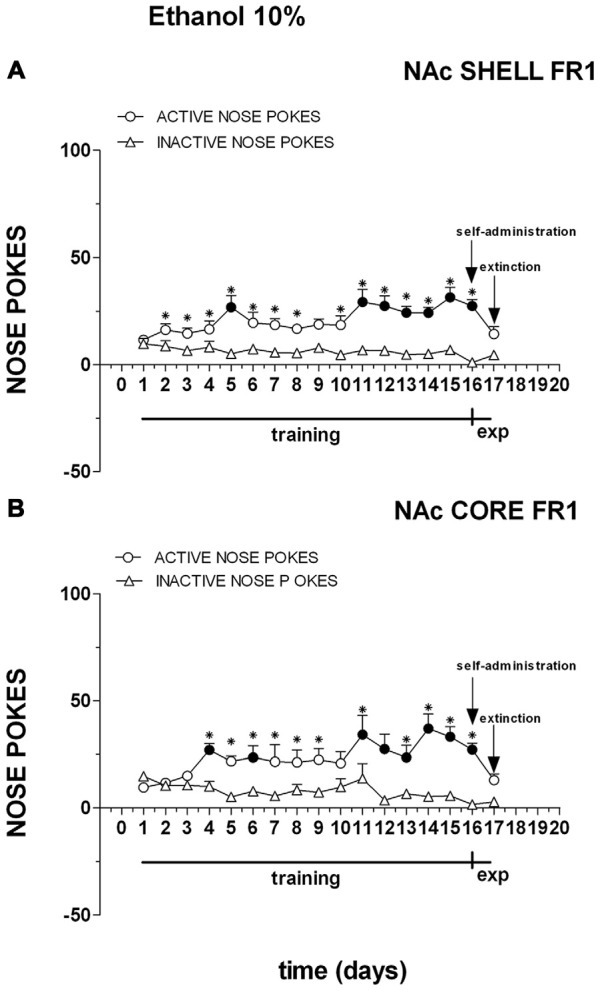
**Cumulative active (circles) and inactive (triangles) nose-pokes during training for FR1 responding for 10% ethanol solution and during dialysis experiments.** Data are means ± SEM of the results obtained in rats implanted with guide cannulas in the NAc shell **(A)** (*N* = 7) and rats in the NAc core **(B)** (*N* = 7). Filled symbols, *p* < 0.05 vs. 1st day; *p < 0.05 vs. inactive nose pokes.

Figure 8 shows the number of cumulative active and inactive nose pokes performed during training for FR1 responding for 10% ethanol, and on the first and the second experiment days. Nose-poking increased selectively on the active hole.

Three-way ANOVA showed a main effect of nose poke (*F*_(1,22)_ = 54.9; *p* < 0.01), days (*F*_(16,352)_ = 3.5; *p* < 0.01) and a nose poke × days interaction (*F*_(16,352)_ = 47.3; *p* < 0.01). Tukey’s test revealed no differences in the temporal profile of acquisition between the shell- and core-implanted groups.

**Figure 9:**
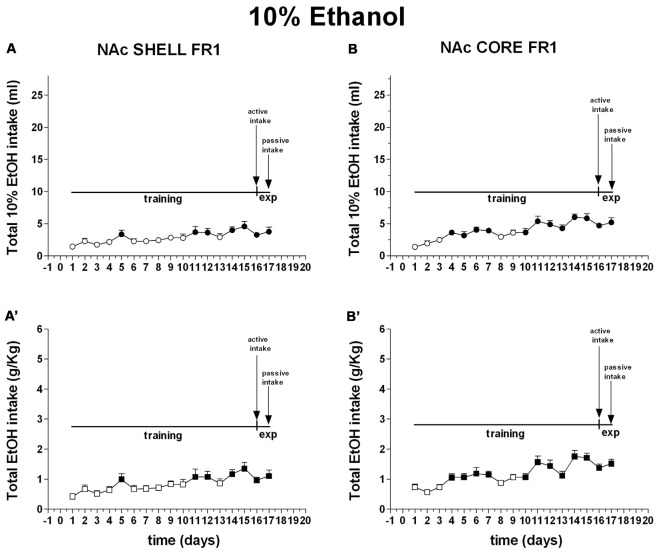
**Total intake expressed as ml of 10% ethanol solution and g/Kg of ethanol.** Data are means ± SEM of the results obtained in rats implanted with guide cannulas in the NAc shell (**A**:ml; **A′**:g/Kg; *N* = 7) and rats in the NAc core (**B**:ml; **B′**:g/Kg; *N* = 7). Filled symbols, *p* < 0.05 vs. 1st day.

Two-way ANOVA of the amount of solution (ml) consumed by rats showed a main effect of days (*F*_(16,160)_ = 4.9; *p* < 0.01). *Post hoc* test revealed that the amount of 10% ethanol solution (ml) consumed by rats increased selectively during the training period but did not reveal any difference between shell- and core-implanted rats (Figure 9).

Two-way ANOVA of the amount of ethanol (g/Kg) consumed by rats showed a main effect of days (*F*_(16,160)_ = 4.2; *p* < 0.01). *Post hoc* test revealed that the amount of ethanol (g/Kg) consumed by rats increased selectively during the training period but did not reveal any difference between shell- and core-implanted rats (Figure 9).

#### Operant Session

**Figure 10:**
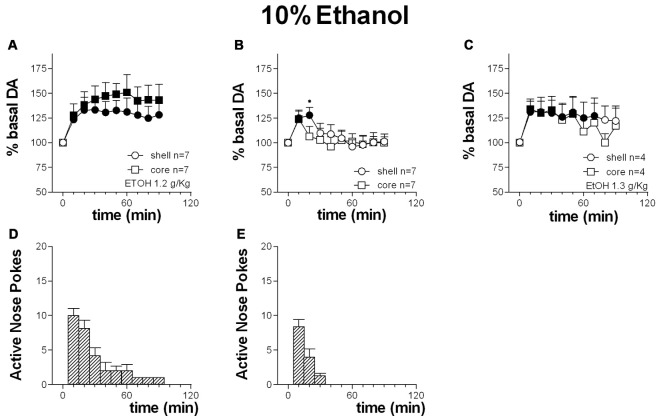
**Time-course of dialysate DA in the NAc shell (circles) and core (squares) and active nose pokes (Bars) every 10 min under FR1 responding for 10% ethanol solution (A,D)**, extinction **(B,E)** and non-contingent 10% ethanol solution presentation **(C)**. Data are means ± SEM of the results, expressed as % of basal values, obtained in the number (*N*) of rats indicated in the figure. Filled symbols: *p* < 0.05 vs. basal values; *p < 0.05 vs. values obtained in the core.

Figures 10A,D shows the time-course of dialysate DA in the NAc shell and core and the number of active nose-pokes performed during FR1 responding for 10% ethanol solution.

Responding for 10% ethanol solution increased dialysate DA to a similar extent in both the shell and the core. Responding remained high for the first 30 min.

Two-way ANOVA showed an effect of area (*F*_(1,12)_ = 7.9; *p* = 0.02), and time (*F*_(9,108)_ = 14.9; *p* < 0.01). *Post hoc* Tukey’s test revealed no differences between the two groups of animals.

#### Extinction Session

Figures 10B,E shows the time-course of dialysate DA in the NAc shell and core and of active nose-pokes under extinction in the presence of cues signaling ethanol availability and associated to ethanol delivery.

Responding under extinction was associated to an increase of DA in both the shell and the core. Responding was high over the first 20 min.

Two-way ANOVA showed an effect of area (*F*_(1,12)_ = 5.4; *p* = 0.04), time (*F*_(9,108)_ = 39.2; *p* < 0.01) and an interaction area × time (*F*_(9,108)_ = 7.7; *p* < 0.01). *Post hoc* test revealed a higher increase in DA in the NAc shell than in the core.

#### Non-Contingent Presentation

Figure 10C shows the time-course of DA in the NAc shell and core following non-contingent presentation of 10% ethanol solution.

Non-contingent ethanol presentation was associated with an increase of dialysate DA both in the NAc shell and core.

Two-way ANOVA showed an effect of area (*F*_(1,10)_ = 6.5; *p* = 0.03) and of time (*F*_(9,90)_ = 10.1; *p* < 0.01). *Post hoc* test did not reveal any differences between the two areas.

## Discussion

This study investigated by microdialysis the responsiveness of NAc shell and core DA transmission to 10% ethanol solution compared to 10% ethanol + 20% sucrose and 20% sucrose solutions; each solution was administered under three different conditions (response-contingent, under extinction trial and response non-contingent) in rats previously trained to respond on a continuous reinforcement (FR1) schedule. An important difference between the present study and previous studies (Weiss et al., 1993; Gonzales and Weiss, 1998; Doyon et al., 2003, 2005; Howard et al., 2009) is that basal levels of dialysate DA were obtained in the Skinner box in which microdialysis was performed. This condition enabled us to avoid the potential artifact of transfer from the home cage to the Skinner box, as in the case of previous studies in which basal samples had been collected in the home cage. Under these conditions, responding for 20% sucrose solution was associated with a selective increase of DA in the shell, while responding for ethanol and ethanol + sucrose solutions was associated with increased dialysate DA both in the shell and core. The same qualitative pattern of changes in DA transmission was obtained in extinction trials. Conversely, response non-contingent presentation of sucrose and ethanol solutions was associated with an increase of DA both in the NAc shell and core. These results provide the first unequivocal evidence that oral ethanol SA increases DA transmission in the rat NAc.

### 20% Sucrose

In rats fully trained to respond for 20% sucrose solutions under a continuous schedule of reinforcement (FR1), dialysate DA increased maximally in the NAc shell over the first 20 min and then decreased, returning to baseline levels by the 4th sample (40 min). In contrast, responding remained high throughout the entire session (60 min), in agreement with our previous observations in rats responding for sucrose pellets (Bassareo et al., 2015c). Thus, in both studies, after an initial surge, the increase in dialysate DA observed in the NAc shell subsequently returned to basal values despite sustained responding, thus becoming dissociated from instrumental action and from the actual intake of sucrose. No change in dialysate DA was observed in the NAc core, again in agreement with our previous observations with sucrose pellets (Bassareo et al., 2015c).

This study also extends to sucrose solutions our previous finding that in instrumentally trained rats, non-contingent presentation and feeding of sucrose pellets (Bassareo et al., 2015c) increases DA in the shell in spite of repeated exposure to sucrose. This finding has been taken to indicate that training to respond for sucrose induces a loss of the habituation of NAc shell DA responsiveness observed in naive rats following response non-contingent sucrose presentation and feeding (Bassareo et al., 2015c). We argued, however, that the loss of habituation of DA responsiveness to sucrose feeding in rats trained for sucrose responding is only apparent, being due to the fact that in these rats NAc shell DA transmission is driven by sucrose discriminative/conditioned stimuli (DS/CS) rather than by sucrose unconditioned stimulus (US; Bassareo et al., 2015c). This hypothesis is based on the assumption that in fully trained rats, the sucrose US is fully predicted by DS/US stimuli, and therefore its DA-stimulant property is lost and transferred to the DS/US stimuli (Schultz et al., 1997). This interpretation, in turn, is consistent with the observation that a similar, although shorter lasting, increase of DA is observed in the NAc shell under an extinction trial, a condition in which sucrose US is absent but DS/CS cues are still present (Bassareo et al., 2015a, c and present study). These observations indicate that the pattern of DA responsiveness in the NAc shell and core compartments to sucrose is strongly dependent on response contingency; specifically, in rats responding by nose-poking, the response of NAc core DA, while allowed under non-contingent sucrose, seems actively suppressed under response-contingent sucrose presentation and feeding.

On the other hand, adaptive modulation of NAc core DA responsiveness depends on the nature of the motor response itself. Indeed, in rats trained to respond for sucrose pellets by lever pressing instead of nose poking, we recently observed that instrumental responding, as well as extinction, was associated with an increase in dialysate DA in both the NAc shell and core. As nose-poking is a rodent-specific, innate motor response, while lever pressing is an unnatural response acquired as a result of training (skill), we suggested that NAc core DA is required to achieve lever pressing but not nose-poking (Bassareo et al., 2015b). Accordingly, whilst NAc core DA may be useful in responding by lever pressing, in the case of nose-poking it is not required, and may even prove counterproductive, potentially inducing “vicious” behavior (stereotypies) that interferes with goal-directed action (Bassareo et al., 2015c), and is therefore suppressed. This hypothesis is consistent with evidence that the NAc shell DA is involved in the inhibition of inappropriate responses in goal-directed action (Ambroggi et al., 2011). Thus, in a context that signals alcohol unavailability, inactivation of NAc shell, rather than inhibiting responding, actually increases it (Chaudhri et al., 2008).

### Responding for 10% Ethanol + 20% Sucrose and for 10% Ethanol

Responding for ethanol, either alone or in association with sucrose, was efficiently acquired, as indicated by the low rate of responding on the inactive compared to the active hole, and by the rapid extinction of responding when ethanol solutions were substituted with water. The rate of responding for 10% ethanol in 20% sucrose was higher than that observed for 10% ethanol alone, but lower than for 20% sucrose. Responding for ethanol alone was maximal for the first two 10 min fractions and then progressively decreased, reaching very low levels in the last 10 min fraction of the session.

With regard to the changes in dialysate NAc DA, a series of differences were observed in rats self-administering ethanol solutions compared to rats responding for sucrose. In rats responding for ethanol solutions, dialysate DA increased in both the shell and core, while in rats responding for sucrose the increase was selective in the shell. On the other hand, similarly to sucrose, non-contingent ethanol solutions increased DA in both the shell and core.

Moreover, responding for sucrose and ethanol solutions further differed in that, in the case of sucrose, the increase of DA in the NAc returned to basal values during the session, when responding was still high, whilst with ethanol solutions DA increased up to a plateau that was maintained beyond the session, in spite of a within session progressive decrease of responding, as occurred with 10% ethanol. We suggest that this intra-session reduction of responding for ethanol is due to the attainment of a plateau of ethanol concentrations in the brain that reduces the need for further ethanol intake. In the case of ethanol, its direct intracerebral action interacts with indirect, cue-related influences on DA to potentially overcome or amplify these. An extensive presence of these interactions is expected during SA of ethanol + sucrose solutions, owing to the fact that the influence of sucrose on DA transmission is peripheral and largely cue-related. Consistent with this suggestion, following ethanol + sucrose SA the pattern of DA increase in the shell vs. core is actually reversed compared to sucrose alone as, rather than being selective to the shell, the increase of DA is actually higher in the core.

During the extinction trial the time-course of DA paralleled that of responding; thus, DA maximally increased in the shell and core at the first sample and then progressively returned to basal values during the session. Moreover, in the extinction trial, DA response was higher in the shell than in the core, in contrast with observations made during operant responding for ethanol, when DA increased to a similar extent in the NAc shell and core.

Although previous studies investigating the changes in NAc DA transmission under operant responding for ethanol did not distinguish between effects produced in the shell and the core (Weiss et al., 1993; Gonzales and Weiss, 1998; Doyon et al., 2003, 2005), the findings of the present study, reporting a similar increase of dialysate DA in the shell and core allow the comparison between our observations and those of studies making no distinction between the shell and core subdivisions of the NAc.

Our observation of an increase in DA in the shell and core under operant responding for sucrose solutions seems in agreement with the conclusions reached by Weiss et al. (1993); Gonzales and Weiss (1998); Doyon et al. (2003, 2005), on the basis of microdialysis studies that made no distinction between shell and core. However, in the above studies, attribution of the increase in NAc DA to ethanol SA is uncertain. Indeed, as previously mentioned in the Introduction, in the studies quoted, basal levels of dialysate DA were obtained from samples collected in the home cage, while dialysate samples of the operant session were collected in the Skinner boxes after a waiting period of 15–20 min. Transfer of rats trained to respond for ethanol solutions from their home cage to the SA cage, or even simple transfer of naive rats from their home cage to a new cage, by itself increases dialysate DA in the NAc (Weiss et al., 1993; Gonzales and Weiss, 1998; Doyon et al., 2003, 2005; Howard et al., 2009). As discussed by Gonzales and Weiss (1998) and by Gonzales et al. (2004) this transfer-induced increase of NAc DA might be due, depending on the conditions, to the acquisition of reinforcer-predictive properties by the SA cage or to the unconditioned incentive properties of novelty. Whatever the mechanism, the transfer-induced rise of DA is a potential confound of any increase in DA during ethanol SA. Admittedly, as pointed out by Gonzales and Weiss (1998), the presence of a similar “artifact” makes it difficult to establish whether, and to what extent, the increase of DA above basal levels is related to ethanol or ethanol + sucrose SA or rather to the combined effect of cage transfer and ethanol SA. In the studies published by Weiss et al. (1993) and by Gonzales and Weiss (1998), the increase in dialysate DA in the NAc is maximal in the first sample after transfer and progressively fades as the rat becomes acclimatized to the test cage. However, the habituation time (wait) before starting ethanol SA after transfer (15–20 min.) might be insufficient to allow DA levels to return to baseline. For example, according to Howard et al. (2009), cage transfer increases DA by 20%–30% in the shell and core, with the increase lasting for at least 15 min. This might explain the failure to observe an increase in DA in the NAc shell and core in rats responding for ethanol + sucrose (Howard et al., 2009).

In the present study, dialysate samples were taken directly in the Skinner box in which operant sessions were performed, and mean levels of DA obtained from three consecutive samples, and differing by no more than 10%, were taken as basal values. These conditions obviated the influence of cage transfer and allowed for full stabilization of DA, thus providing appropriate basal reference levels of dialysate DA.

In the article, Howard et al. (2009) reported that ethanol SA increases dialysate DA in an area of the NAc that they call the “shore” (see “Introduction” Section), but not in the shell nor in the core. The “shore”, however, is a virtual area, comprised of the shell and core tissue located along their adjoining border. Accordingly, microdialysis probes placed in this area would recover DA, depending on the precise location, from the shell and, respectively, from the core. In view of this, a more parsimonious interpretation of the results by Howard et al is that ethanol increases dialysate DA both in the shell and in the core, in agreement with our observations.

Our observations might also be discussed in terms of the nature of changes in dialysate DA observed following exposure to ethanol. Doyon et al. (2003, 2005) suggested that changes in dialysate DA in rats responding for ethanol are not the result of a direct central effect of ethanol since their peak precedes that of ethanol in the blood. This suggestion is consistent with our previous observations that in rats given ethanol through intraoral cannulas, the increase of DA in the NAc shell is biphasic, with an initial peak related to the taste of ethanol and a second peak coincident with the rise of ethanol in dialysates (Bassareo et al., 2003). These observations, however, were made in rats naive to the taste of ethanol, in which habituation to the taste-induced activation of DA is observed. In an operant paradigm ethanol taste might undergo habituation as a primary US and be converted into a CS as a result of instrumental conditioning. As long as it is reinforced, taste CS-induced increase of DA in the NAc shell should not habituate, thus explaining the apparent loss of habituation of DA responsiveness in rats given ethanol solutions non-contingently. Consistent with a role of non-taste ethanol-conditioned CSs, dialysate DA increases in both the shell and core in extinction trials, when ethanol is substituted by water. Similar observations have been made under extinction from sucrose pellets, in which case, however, they were selective to the shell (Bassareo et al., 2015c and present study).

Due to the limited temporal resolution of microdialysis techniques, the relative contribution of non-taste DS/CS preceding taste US/CS to the increase in dialysate DA in rats responding for ethanol solutions cannot be ascertained. It is however clear that with both ethanol and sucrose, the changes in dialysate DA obtained during the extinction trial follow the same shell/core pattern as during operant sessions; this finding suggests that in instrumentally trained rats discriminative CS are *per se* sufficient to account for the changes in dialysate DA that occur during operant sessions.

From this point of view ethanol differs from other drugs of abuse. Thus, while DA remains flat during extinction trials from heroin, cocaine, nicotine and Win 55,212-2 SA both in the shell and core (Lecca et al., 2006a,b, 2007a,b), it increases in both shell and core under responding for ethanol. In our opinion, these major differences are linked to the fact that ethanol is administered orally and to the acquisition of taste as a highly salient secondary reinforcer. Thus, once the predictive association between the taste of ethanol and its systemic effects has been made, rats work for ethanol taste, a mechanism that likely applies also to sucrose reinforcement. With training, instrumental conditioning also generalizes to non-taste cues, thus explaining the increase of dialysate DA under extinction trials, in the absence of taste cues. A notable difference between sucrose and ethanol reinforcement is related to the pattern of DA responsiveness in the shell and core under operant responding and extinction. Indeed, on exposure to sucrose a similar pattern of response is observed in the two conditions, since DA increases selectively in the shell; conversely, in the presence of ethanol + sucrose, DA increases preferentially in the core during reinforced, and in the shell during non-reinforced, sessions. Once again, this might be due to the action of ethanol, which activates DA neurons in reinforced sessions and thus overcomes the influence of other cues on DA transmission.

These observations seem to suggest that the differences observed between ethanol and sucrose are partly due to the fact that ethanol similarly activates NAc DA in the shell and in the core; this in turn distinguishes ethanol from other drugs of abuse that preferentially activate DA in the shell after i.v. SA (Lecca et al., 2006a,b, 2007a,b). It is however unclear whether the differences observed are due, and to what extent, to the use of diverse routes of administration for ethanol (oral) and for the other drugs (i.v.).

The ability of ethanol SA to activate DA in the NAc core, and the ability of sucrose to activate DA selectively in the shell, may underlie the differential roles played by NAc shell and core DA in paradigms of pavlovian to instrumental transfer (PIT), recently reported by Corbit et al. (2016). PIT consists in the ability of a conditioned stimulus (CS) previously paired to a given US, to invigorate responding for the same (outcome-selective PIT) or for a different US (general PIT; Corbit et al., 2007). The two forms of PIT are differentially dependent on the integrity of the two subdivisions of the NAc. While general PIT depends on the NAc core, outcome-selective PIT depends on the shell (Corbit et al., 2007). Similarly, while ethanol-paired stimuli increase responding both for ethanol on the ethanol lever and for sucrose on the sucrose lever (general PIT), sucrose paired stimuli increase responding on the sucrose but not on the ethanol lever (outcome-selective PIT), with these effects being selectively impaired by inactivation of the core and, respectively, of the shell (Corbit et al., 2016). We suggest that these differences are related to the differential pattern of activation of NAc shell and core DA during instrumental performance, that differentially interacts with the incentive properties of pavlovian stimuli conditioned to ethanol and sucrose, respectively.

## Conclusions

In conclusion, the present study, provides clear evidence unconfounded by the influence of transfer from the home cage to the SA box, that oral SA of a 10% ethanol solution is associated with a rapid activation of DA transmission in the NAc shell and core that reaches a plateau and is maintained throughout the session despite a progressive decrease in responding following the initial surge. Comparison of the pattern of activation of DA transmission following the administration of sucrose, sucrose + ethanol and ethanol solutions highlights the existence of important differences between sucrose and ethanol rewards, suggesting that the different patterns obtained are the result of a complex interaction between the direct intracerebral action of ethanol and the action of peripheral stimuli arising from sucrose and ethanol USs and from their related DS/CSs. A key aspect in the pattern of DA transmission following exposure to an ethanol solution is the activation of NAc core DA, which contrasts with the selective activation of NAc shell DA by sucrose SA; this in turn is at variance with the combined activation of shell and core DA following passive sucrose presentation and consumption. These observations suggest that major adaptive mechanisms, such as active inhibition of NAc core DA transmission, are activated during instrumental responding for sucrose. These mechanisms may be related to a basic function of NAc shell DA, that of preventing the expression of changes (e.g., increase of NAc core DA) inappropriate for correct goal-directed action. Conversely, following exposure to ethanol, the direct action of ethanol and the resulting increase in NAc core DA may be amplified by its stimulus properties or by DS/CS cues. This in turn would result in a general motivational arousal, overcoming fine adaptive mechanisms and resulting in a peculiar behavioral abnormality whereby pavlovian stimuli unrelated to ethanol are nonetheless capable of invigorating ethanol seeking and consumption, much like the general transfer effect displayed in experimental PIT paradigms. Such generalized activation might contribute to the abnormal features of ethanol reward as compared to a conventional one like sucrose.

## Author Contributions

VB: study design; acquisition, analysis and interpretation of data; draft of the article. FC: acquisition and analysis of data. RF: acquisition and analysis of data. GDC: study design, theoretical elaboration of the data, draft and final revision of the article.

## Funding

This work was supported by grants from Fondazione Banco di Sardegna (2011.1047. Prot n.U1140.2013/AI.1059.MGB).

## Conflict of Interest Statement

The authors declare that the research was conducted in the absence of any commercial or financial relationships that could be construed as a potential conflict of interest.
